# From Genes to Ecosystems in Microbiology: Modeling Approaches and the Importance of Individuality

**DOI:** 10.3389/fmicb.2017.02299

**Published:** 2017-11-27

**Authors:** Jan-Ulrich Kreft, Caroline M. Plugge, Clara Prats, Johan H. J. Leveau, Weiwen Zhang, Ferdi L. Hellweger

**Affiliations:** ^1^Centre for Computational Biology, Institute for Microbiology and Infection, School of Biosciences, University of Birmingham, Birmingham, United Kingdom; ^2^Laboratory of Microbiology, Wageningen University and Research, Wageningen, Netherlands; ^3^Department of Physics, School of Agricultural Engineering of Barcelona, Universitat Politècnica de Catalunya–BarcelonaTech, Castelldefels, Spain; ^4^Department of Plant Pathology, University of California, Davis, Davis, CA, United States; ^5^Laboratory of Synthetic Microbiology, Key Laboratory of Systems Bioengineering (Ministry of Education), School of Chemical Engineering and Technology, Tianjin University, Tianjin, China; ^6^Civil and Environmental Engineering Department, Marine and Environmental Sciences Department, Bioengineering Department, Northeastern University, Boston, MA, United States

**Keywords:** microbial ecology, gene-centric modeling, metabolic flux modeling, agent-based modeling, individuality, heterogeneity, single cell

## Abstract

Models are important tools in microbial ecology. They can be used to advance understanding by helping to interpret observations and test hypotheses, and to predict the effects of ecosystem management actions or a different climate. Over the past decades, biological knowledge and ecosystem observations have advanced to the molecular and in particular gene level. However, microbial ecology models have changed less and a current challenge is to make them utilize the knowledge and observations at the genetic level. We review published models that explicitly consider genes and make predictions at the population or ecosystem level. The models can be grouped into three general approaches, i.e., metabolic flux, gene-centric and agent-based. We describe and contrast these approaches by applying them to a hypothetical ecosystem and discuss their strengths and weaknesses. An important distinguishing feature is how variation between individual cells (individuality) is handled. In microbial ecosystems, individual heterogeneity is generated by a number of mechanisms including stochastic interactions of molecules (e.g., gene expression), stochastic and deterministic cell division asymmetry, small-scale environmental heterogeneity, and differential transport in a heterogeneous environment. This heterogeneity can then be amplified and transferred to other cell properties by several mechanisms, including nutrient uptake, metabolism and growth, cell cycle asynchronicity and the effects of age and damage. For example, stochastic gene expression may lead to heterogeneity in nutrient uptake enzyme levels, which in turn results in heterogeneity in intracellular nutrient levels. Individuality can have important ecological consequences, including division of labor, bet hedging, aging and sub-optimality. Understanding the importance of individuality and the mechanism(s) underlying it for the specific microbial system and question investigated is essential for selecting the optimal modeling strategy.

## Introduction

Microbes are important drivers of biogeochemical cycles in all ecosystems and impact their environments in a plethora of ways. For example, in lakes, the harmful cyanobacterium *Microcystis aeruginosa* can bloom and produce toxins that make the water unsafe to drink (Paerl et al., 2011). The common gut bacterium *Bacteroides fragilis* produces a chemical that helps the host develop its immune system (Atarashi et al., 2013).

Models are important tools for understanding and managing ecosystems. They can be used to advance scientific understanding by interpreting field observations and aid in hypothesis testing. For example, Jöhnk et al. (2008) used a model to quantify the roles of temperature range and buoyancy regulation in the fitness of the toxic cyanobacterium *Microcystis* during heat waves. Buffie et al. (2015) applied the model of Stein et al. (2013) to infer an antagonistic interaction in the gut between the pathogen *Clostridium difficile* and another species of that genus, *Clostridium scindens*. For ecosystem management, models can be used to answer “what if” questions and make predictions about the effects of future environmental conditions. For example, Blumberg and Di Toro (1990) used a model to predict the effects of climate warming on phytoplankton and dissolved oxygen in a lake. Bucci et al. (2016) predicted the composition of the mouse gut microbiota following infection with *C. difficile*.

In the past decades, microbiology has experienced rapid advances in observational and experimental technologies, resulting in substantial progress in the understanding of microbes at the molecular level. For example, nitrogen (N) fixation by the cyanobacterium *Anabaena* involves a division of labor among N-fixing heterocysts and photosynthesizing vegetative cells. The nitrogen-containing β-aspartyl-arginine is produced by cyanophycinase in heterocysts, transferred intercellularly to vegetative cells where it is converted to aspartate and arginine by isoaspartyl dipeptidase (Burnat et al., 2014). Another example involves transcription of genes to messenger RNA (mRNA) and translation to proteins, which is performed by RNA polymerase (RNAP) and the ribosome complex, respectively. In bacteria, those can form a single transcribing and translating “expressome” complex, with known 3D structure and functional consequences on transcriptional pausing, backtracking and termination (Kohler et al., 2017). Characterization of ecosystems is following the same trend. For example, lakes used to be characterized using bulk measures, like Chlorophyll *a* and total phosphorus concentrations, but observations are now often at the molecular level, including gene expression (transcript levels) (Vila-Costa et al., 2013). Animal and human microbiota are now routinely characterized using multiple omics technologies, such as community characterization using bacterial 16*S* ribosomal RNA (rRNA) polymerase chain reaction (Costello et al., 2009), and increasingly meta-genomics, transcriptomics and proteomics (Wang et al., 2015).

The development of models is lagging behind as most models still do not make use of molecular level understanding or observations. It is recognized that there is a substantial gap between our microbial ecology models and current microbiology knowledge and environmental observations (Fuhrman et al., 2013; Trivedi et al., 2013; Hellweger, 2015; Dick, 2017; Stec et al., 2017). For example, lake phytoplankton models still simulate phytoplankton biomass concentrations (e.g., μg Chlorophyll *a* L^−1^) and the effect of a nutrient on the growth rate using an equation developed 75 years ago (Monod model). Likewise, most models of the gut aggregate species into functional groups based on metabolic pathways (Kettle et al., 2015). Models are now being developed that explicitly resolve genes and make predictions at the population and ecosystem level.

This paper has two parts. First, we review existing modeling approaches. Here, we focus on mechanistic models that explicitly include genes and simulate population-level properties (e.g., microbe concentration, nutrient uptake) rather than empirical models. One aspect in which the existing approaches differ is their representation of microbial individuality. The second part of our review will use examples to explain why including individuality is important.

## Part 1: review of existing modeling approaches

**Figure 1:**
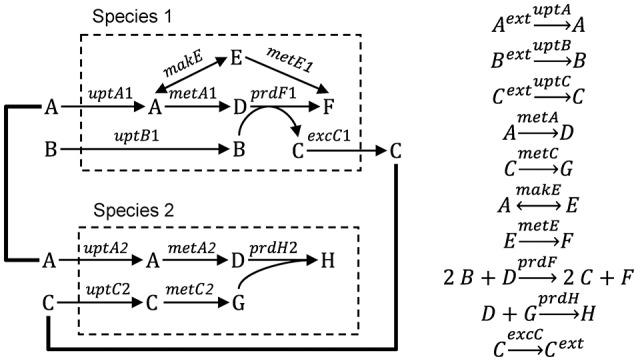
Hypothetical ecosystem used to illustrate different modeling approaches. Species 1 takes up metabolites A and B, produces metabolites C, D, E, and F and excretes metabolite C. Species 2 takes up metabolites A and C and produces metabolites D, G, and H.

In this section we describe three modeling approaches that have been used to bridge the gap between genes and ecosystems, including metabolic flux, gene-centric and agent-based modeling (ABM). We illustrate each approach using a hypothetical ecosystem, where two microbial species grow and interact via three metabolites (Figure 1). We then discuss a number of examples from the literature, focusing mostly on the modeling aspects of the studies. Then we highlight the weaknesses and strengths of each approach. Finally, we characterize the models along a number of dimensions, including space, time, function, heterogeneity, species diversity and genes.

### Literature selection criteria

The review is focused on the use of gene-level models for advancing understanding and making predictions of microbial ecosystems. To keep the scope of the review manageable, we included only quantitative models, which are required for predictions, although qualitative models may be sufficient to advance understanding. We applied the following selection criteria: (1) model uses a mechanistic (vs. empirical) approach, (2) model explicitly considers at least one actual gene or protein; (3) model includes some form of direct or indirect interaction among microbes; (4) model includes multiple microbial species (or strains) or phenotypes in different locations; and (5) model makes predictions at the population level. We therefore exclude empirical models that correlate observed gene distributions to environmental factors and function (e.g., carbon export in the ocean, Guidi et al., 2016), models that use hypothetical genes or digital genomes describing behavioral traits (e.g., Lenski et al., 1999; Clark et al., 2011), scale up single-cell models using multiple independent simulations where the cells do not interact (e.g., Emonet and Cluzel, 2008; Labhsetwar et al., 2013) and studies that infer interactions from comparison of metabolic networks and do not make quantitative predictions (e.g., Levy and Borenstein, 2013; Zelezniak et al., 2015).

### Metabolic flux modeling

#### Definition

This approach builds on the genome-scale, constraint-based modeling approach most commonly applied to single species (Feist et al., 2008). In this approach, the genome sequence is used to derive a network of potential metabolic reactions by a combination of automated and manual (curation) steps. Then, a flux distribution is predicted, typically by optimizing the flux distribution to maximize an objective function, like maximization of biomass production (Schuster et al., 2008). The extension of this approach to multiple species builds on efforts to extend it to multiple compartments of higher eukaryotic organisms. There are three approaches to multi-species metabolic flux modeling, which we will refer to as environmentally coupled, directly linked and aggregated approaches. The environmentally coupled approach builds on the dynamic flux balance analysis (FBA) approach (Varma and Palsson, 1994), where the microbes and extracellular metabolites are represented using concentration state variables. The growth rate and metabolite fluxes are computed from FBA assuming a common pool for extracellular metabolites and that the system is in a steady state during each time step. The directly linked approach explicitly links the metabolic networks of the species using exchange reactions. This is conceptually the same way in which multi-compartment organisms are modeled. The aggregated approach (also referred to as pooled, supra-organism or enzyme soup approach) involves constructing one network by combining the individual networks and removing duplicates. This ignores cellular boundaries and is most applicable to metagenomic datasets. Box 1 illustrates these three approaches for the hypothetical ecosystem shown in Figure 1. This approach has also been referred to as Ecosystems Biology (Klitgord and Segrè, 2011) or Community Systems Biology (Zengler and Palsson, 2012).

Box 1 **From genes to ecosystems using metabolic flux modeling.**

Single species

The starting point for a metabolic flux model is a set of mass balance equations:

(B1.1) $$\begin{array}{l} \frac{d\text{x}}{dt}=\text{S}\cdot \text{v} \end{array}$$

where ***x*** (mmol gDW^−1^, i.e., per gram biomass dry weight) is a vector of metabolite concentrations, ***S*** is the stoichiometric matrix and ***v*** (mmol gDW^−1^ h^−1^) is a vector of reaction rates for uptake, excretion, internal metabolism and growth. Typically, a steady-state is assumed so the derivatives are zero. The stoichiometric matrix (***S***) for species 1 of the hypothetical ecosystem is presented in Table B1, where columns are reactions and rows are metabolites. Lower and upper bounds for the reaction rates, determined based on thermodynamics, enzyme kinetics or measurements, can be included in the optimization procedure.

**Figure B1.1:**
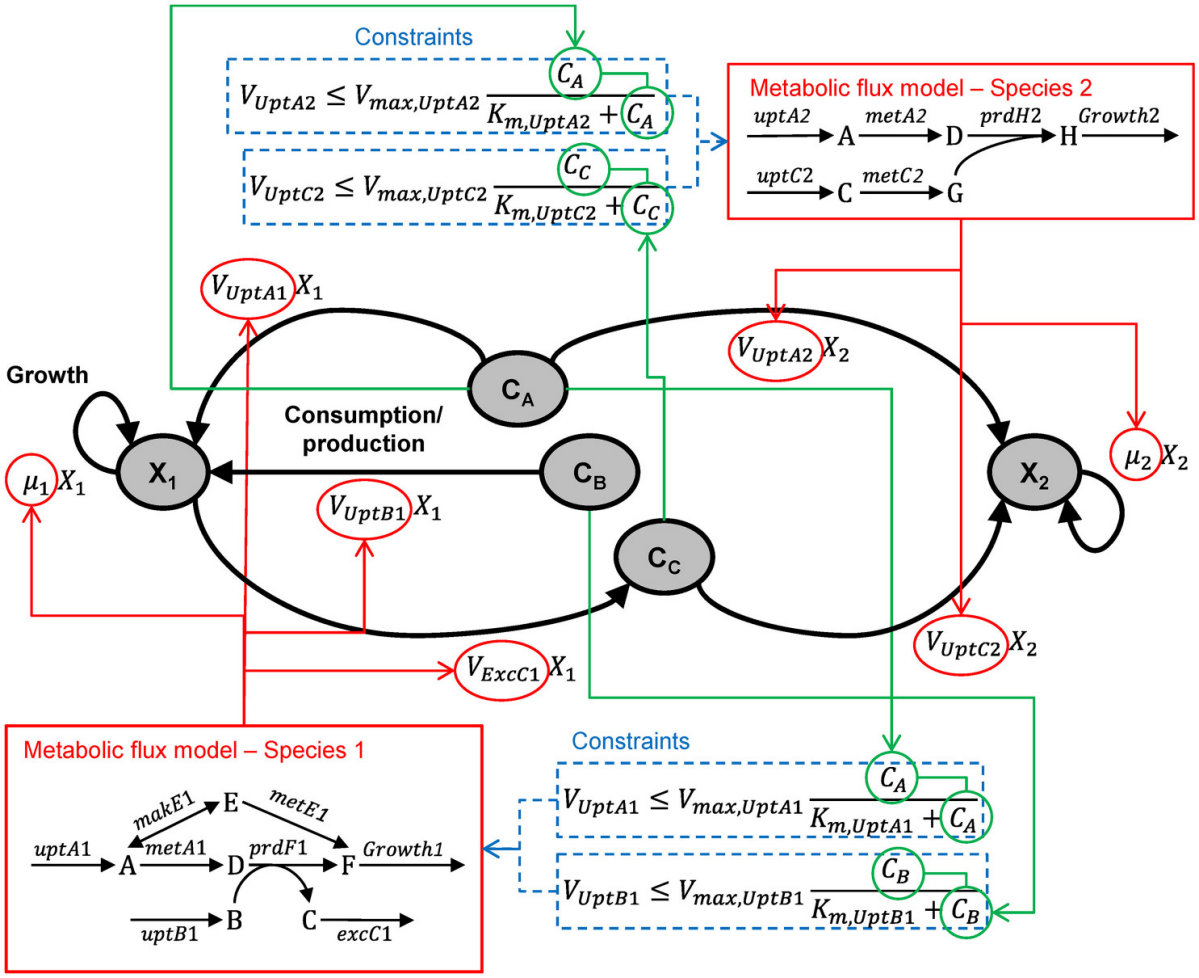
Multi-species metabolic flux modeling—environmentally coupled models. After Figure 2 in Zhuang et al. (2011). *X* (gDW L^−1^) = microbial biomass concentration, C (mmol L^−1^) = extracellular metabolite concentrations, μ (h^−1^) = specific growth rates, *V* (mmol gDW^−1^ h^−1^) = specific flux velocities.

There are typically infinitely many solutions that satisfy the equation. For example, in species 1 (Figure B1.1), biomass (metabolite F) can be produced by any combination of two pathways (A
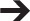
E
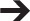
F or A
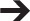
D
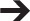
F). Computational methods are available that decompose the stoichiometric matrix into unique sets of functional units (pathways) such as elementary modes or extreme pathways (Papin et al., 2004). A more common approach, flux-balance analysis (FBA), involves optimizing reaction rates to maximize the value of some objective function using linear programing (LP). Several objective functions have been used, such as minimizing ATP production and maximizing production of some metabolite, but maximizing biomass production yield or rate is often considered to be the most appropriate in an ecological context. When biomass production is maximized, it is assumed that the cell regulates fluxes through its metabolic network in a way that maximizes biomass production. The corresponding objective function for the species 1 of our example system is to maximize the production of metabolite F (*V*_*MetE*1_ + *V*_*PrdF*1_ or *V*_*Growth*1_ in Table B1). This is relatively simple and real models typically use a more complex biomass growth function, e.g., a genome-scale model may include various precursors (e.g., G6P, F6P) and cofactors (e.g., ATP, NADH). Algorithms that integrate gene expression data are also available (Becker and Palsson, 2008). FBA is fundamentally a steady-state approach, but a pseudo-time-variable model can be constructed (Varma and Palsson, 1994; Mahadevan et al., 2002).

Multiple species—environmentally coupled models

Figure B1.1 illustrates the dynamic, multi-species metabolic flux modeling methodology. The model includes state variables for microbial biomass (*X*) and extracellular metabolites (*C*). The microbes grow according to a growth rate (μ) and consume/produce metabolites according to specific consumption/production rates (*V*). Those values are calculated from the metabolic flux models, which are optimized to maximize the growth yield subject to a number of constraints, including a maximum consumption rate for each metabolite based on its concentration. A simulation will proceed in a step-wise manner: (1) Calculate the constraints based on all *C*. (2) Optimize the metabolic model of each species, which yields μ and *V*. (3) Calculate the new *X* for both species based on μ. (4) Calculate the new *C* for both metabolites based on *V* from both species. Repeat. When the metabolic model does not lead to a viable solution, a simple death routine can be invoked (Zhuang et al., 2011). It is conceptually straightforward to include other reactions (e.g., between extracellular compounds) and transport (Scheibe et al., 2009).

Multiple species—directly linked model

**Figure B1.2:**
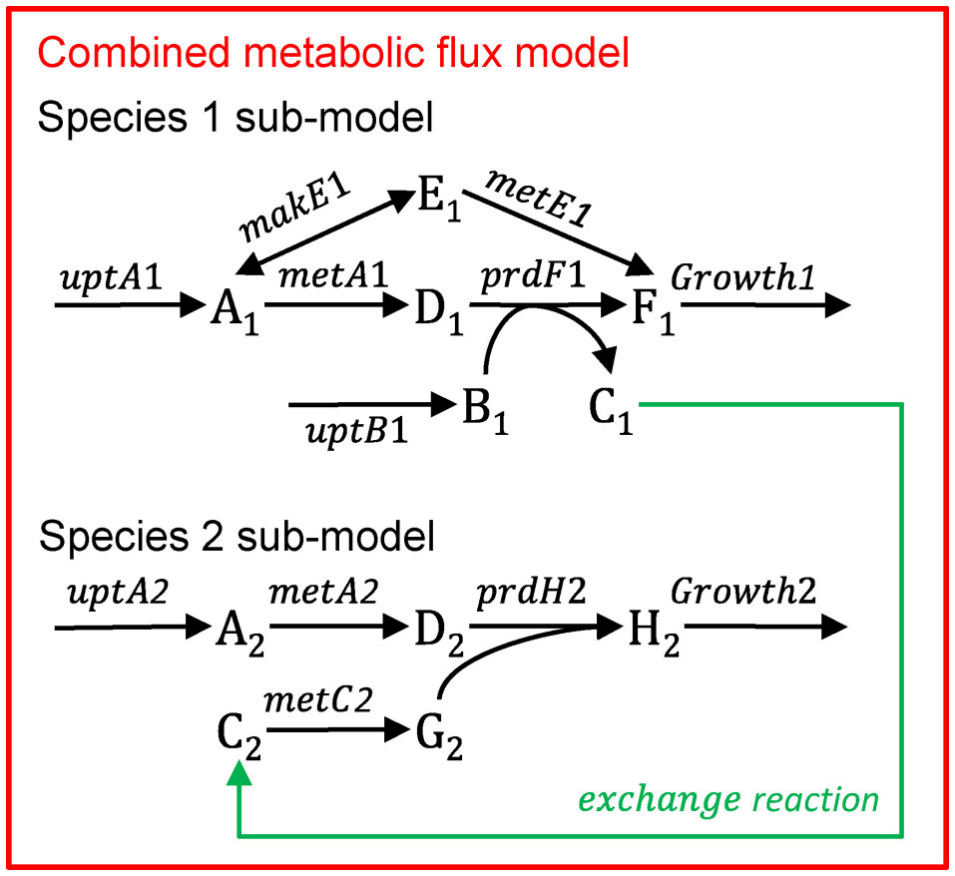
Multi-species metabolic flux modeling—directly linked model. After Figure 2 in Stolyar et al. (2007). The metabolic models for each species (Figure B1.1) are combined into one model, with exchange reactions linking their metabolisms.

Figure B1.2 illustrates the multi-species metabolic flux modeling approach developed by Stolyar et al. (2007). The metabolic models for each species (Figure B1.1) are combined into a single model. Exchange of metabolites among the species occurs by directly linking their reactions, which constrains them to be the same. This is equivalent to assuming there is no change in the extracellular metabolite concentrations. The model is optimized to maximize a weighted combination of the biomasses.

Multiple species—aggregated model

**Figure B1.3:**
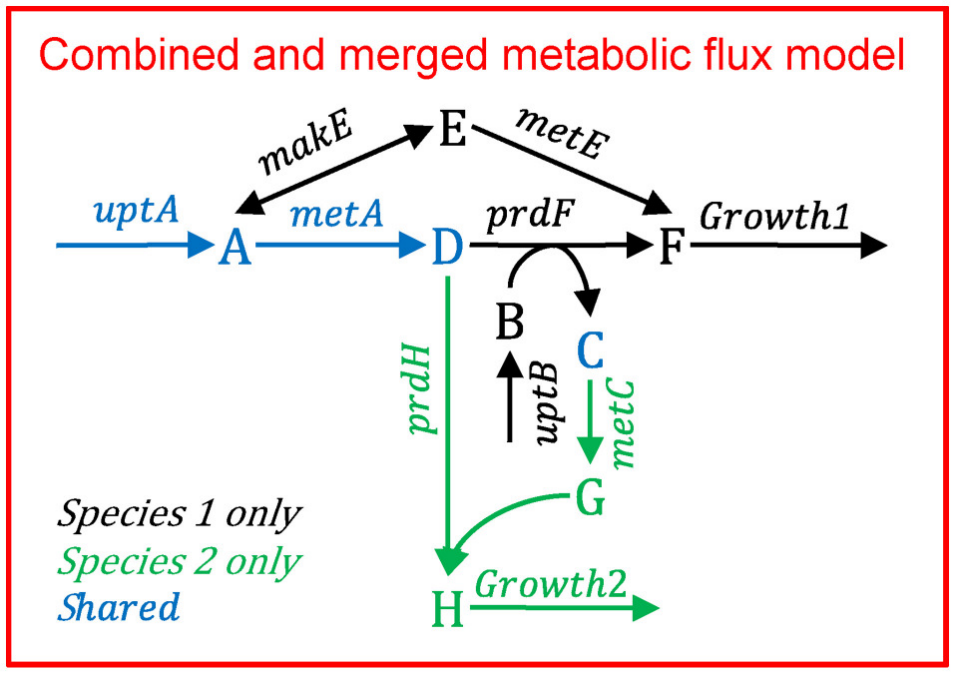
Multi-species metabolic flux modeling—aggregated model. After Figure S2 in Taffs et al. (2009). The metabolic models for each species (as shown in Figure B1.2) are merged into one model.

Figure B1.3 illustrates the multi-species metabolic flux modeling approach developed by Taffs et al. (2009). The reactions and metabolites for the two species (as shown in Figure B1.1) are merged into a single model and a single objective function is used to determine the flux distribution.

**Table B1:** Stoichiometric Matrix (S) and lower and upper bounds for the FBA of species 1.

	***V_*MetA*1_***	***V_*MakE*1_***	***V_*MetE*1_***	***V_*PrdF*1_***	***V_*UptA*1_***	***V_*UptB*1_***	***V_*ExcC*1_***	***V_*Growth*1_***
A	−1	−1	0	0	1	0	0	0
B	0	0	0	−2	0	1	0	0
C	0	0	0	2	0	0	−1	0
D	1	0	0	−1	0	0	0	0
E	0	1	−1	0	0	0	0	0
F	0	0	1	1	0	0	0	−1
Lower	0	– ∞	0	0	0	0	0	0
Upper	∞	∞	∞	∞	MM^*^	MM^*^	∞	∞

Rows are metabolites and columns are reactions.

* *Calculated from extracellular substrate concentration using the Michaelis-Menten function (see Figure B1.1)*.

#### Examples

There have been several applications of metabolic flux models to communities of microbes. For recent reviews see Zengler and Palsson (2012), Biggs et al. (2015), Tan et al. (2015), Zomorrodi and Segrè (2016), Perez-Garcia et al. (2016), and Gottstein et al. (2016).

##### Environmentally coupled models

**Figure 2:**
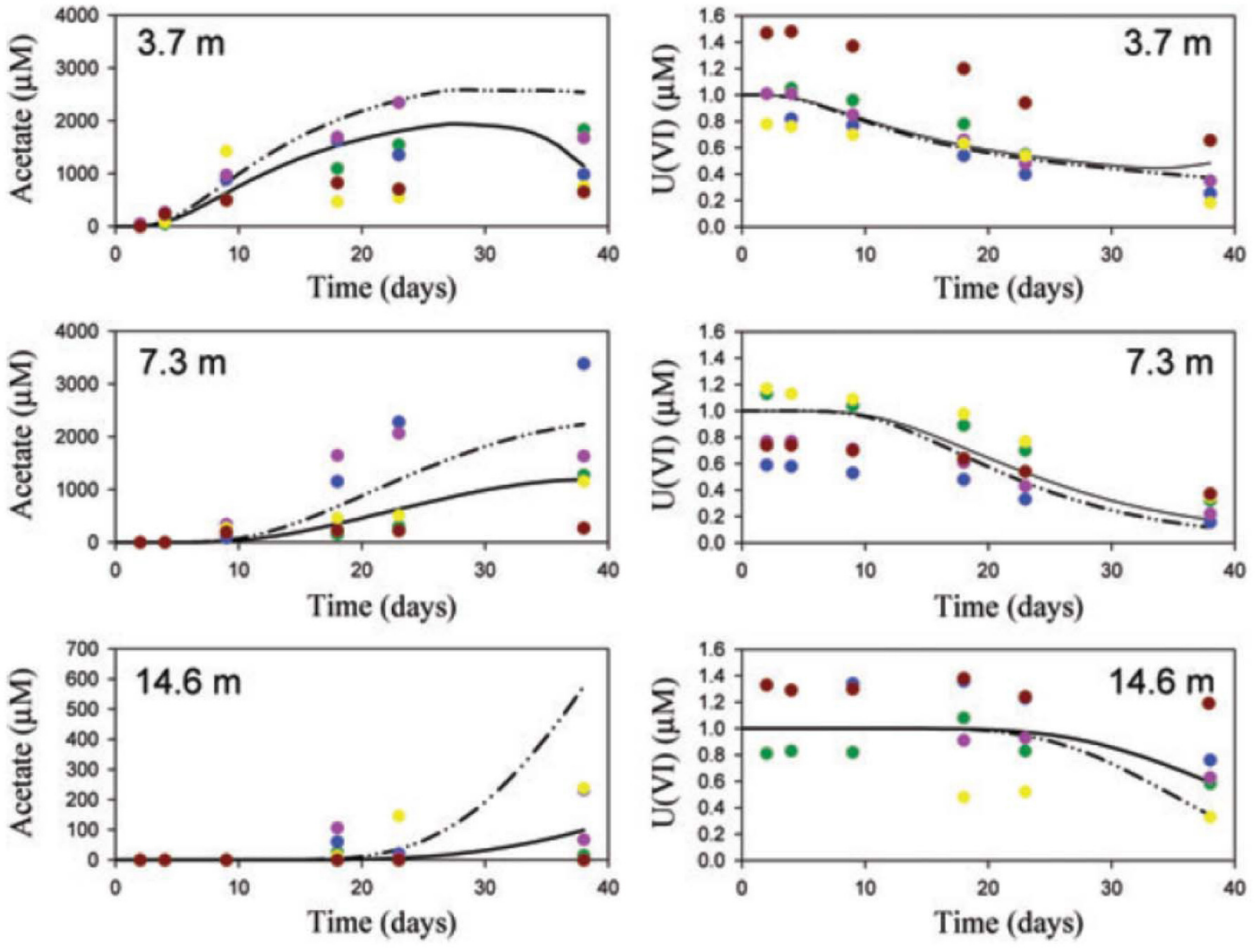
Comparison of observations (symbols), traditional model (solid lines) and FBA-based model (dashed lines). Reproduced from Scheibe et al. (2009) with permission. The figure shows acetate and U(VI) concentrations at a groundwater bioremediation site. Concentration time series are presented at 3.7, 7.3, and 14.6 m distance from the acetate injection gallery. Acetate increases at progressively later times as the distance from the injection gallery increases. Consistent with this, U(VI) decreases at progressively later times. Colors identify single wells.

Scheibe et al. (2009) applied FBA to learn about the growth of *Geobacter* and uranium bioremediation in a contaminated groundwater site where *Geobacter* dominates the community. They coupled a genome-scale FBA model to a two-dimensional reactive transport model. The FBA model computes growth rate and fluxes based on ambient acetate, Fe(III) and ammonia concentrations in each grid element. Those growth rates and fluxes are then used by the reactive transport model to compute the *Geobacter* biomass, acetate, Fe(III) and ammonia concentrations, as well as other processes like U(VI) reduction. The new ambient concentrations are then again used by the FBA model to compute the growth rate and fluxes at the next time step and so on. Due to computational constraints, the FBA calculations were done *a priori* for 1,000 combinations of metabolite concentrations and stored in a look-up table, rather than a dynamic coupling between the models. One of the main advantages of the FBA-based approach is that it allows for variable substrate utilization and growth yields, which is not supported by conventional models. The model was able to make predictions of similar quality as the previous reactive transport model (i.e., without FBA component), but it did so without the need to calibrate rate parameters (Figure 2).

Tzamali et al. (2009) and Tzamali et al. (2011) used the dynamic FBA approach to simulate the interaction among various *E. coli* strains, including wild type and single gene knockouts. For various substrates, they identified potential communities of co-existing strains. For example, growth on pyruvate supported communities with up to 6 strains. The most efficient community of 4 mutants produced 2.2% more biomass than a pure culture of the wild type.

Zhuang et al. (2011) developed a dynamic, genome-scale FBA model of two species in competition in a uranium-contaminated aquifer. *Rhodoferax* and *Geobacter* both oxidize acetate and reduce Fe(III), but only *Geobacter* can reduce U(VI), rendering it less soluble and therefore contributing to the clean-up of the site. The FBA models of the two species calculate growth and metabolite production/consumption rates, which are used to integrate biomass and metabolite concentration state variables. The model predicted that, under low-ammonia conditions, *Rhodoferax* is outcompeted by *Geobacter*, which can fix nitrogen, and that this promotes respiration (vs. biomass production) and associated U(VI) reduction, which are patterns consistent with observations.

Zhuang et al. (2012) expanded the model by Zhuang et al. (2011) and applied it to design remediation scenarios. In particular, they used two separate FBA models for attached and planktonic *Geobacter* to differentiate their functions: planktonic cells reduce U(VI) and attached cells reduce Fe(III). Attachment and detachment rates were used to transfer biomass among these two fractions. This illustrates one approach by which heterogeneity can be simulated in these types of models.

Harcombe et al. (2014) developed dynamic FBA models of two and three species on a two-dimensional grid, where biomass grows and dies, extracellular metabolites are consumed and produced, and biomass and metabolites move by diffusion. Cole et al. (2015) extended the dynamic FBA approach further to three dimensions and used it to simulate growth of *E. coli* in colonies on agar. The model was able to simulate the small-scale environmental heterogeneity in dissolved oxygen and nutrient concentrations, and the resulting phenotypic differentiation of the bacteria (i.e., fermenting cells in the interior). Other multi-species, environmentally coupled metabolic flux models were presented by Salimi et al. (2010), Hanly and Henson (2011), Hanly and Henson (2013), Biggs and Papin (2013), Chiu et al. (2014) and Louca and Doebeli (2015). Zomorrodi et al. (2014) presented a dynamic version of the multi-level optimization routine presented previously (Zomorrodi and Maranas, 2012, see below).

##### Directly linked models

Stolyar et al. (2007) developed an FBA model of two microbes that are mutualistic in the absence of sulfate, *Desulfovibrio vulgaris* and *Methanococcus maripaludis*. In the scenario evaluated, *D. vulgaris* grows on lactate, producing H_2_, formate, CO_2_ and acetate, which support the growth of *M. maripaludis*. The model consists of three compartments, representing the metabolism of the two species and the exchange between them. The metabolite fluxes in the central metabolism of each species and exchange reactions are represented using 89 and 82 equations, respectively. The third compartment represents the exchange flux of H_2_, formate, CO_2_ and acetate, where H_2_ and formate were not allowed to accumulate in the medium, so that their rates of production by *D. vulgaris* and consumption by *M. maripaludis* are the same. The combined model was optimized to maximize biomass production of both species, with a larger weight for *D. vulgaris*, based on observations. However, the biomass ratio of the two species is constrained by the exchange reaction, so it was relatively invariant to the weights used. The model suggested that the H_2_ was essential, but that formate could be eliminated.

Wintermute and Silver (2010) applied the FBA modeling approach at the genome scale to 46 *E. coli* mutants, each incapable to synthesize an essential metabolite. Growth experiments were conducted with 1,035 binary strain combinations. A joint FBA of each pair allowing for exchange of all shared metabolites between the strains was developed. The models were optimized to minimize the difference between the flux distributions of the wildtype and mutant (minimization of metabolic adjustment, MOMA, Segrè et al., 2002). The idea behind this objective function is that the regulatory system is still based on the wildtype and has not yet adjusted to the mutation. The joint FBA models were consistent with the finding that pairings of mutants blocked in the same biosynthetic pathway rarely show synergistic growth (4% of the cases) while pairings of mutants in separate pathways did so in 18% of cases. The model correctly predicted that strains grow best when they require small amounts of metabolites that are cheap to produce by the other strain. The ability of simple stoichiometric models to predict fitness costs and benefits of metabolic cross-feeding is encouraging.

Klitgord and Segrè (2010) applied the FBA modeling approach to binary pairs of seven species and identified the media composition that would support symbiosis. They developed genome-scale FBA models of all possible binary pairs and did a systematic search for media compositions that would support growth of the pair but not the individual species.

Huthmacher et al. (2010) generated an FBA model of the metabolism of the malaria causing *Plasmodium falciparum* and its host, the erythrocyte (red blood cell). By constraining the metabolic network with gene expression data of *P. falciparum*, they were able to predict metabolic fluxes for different life cycle stages of the pathogen.

Zomorrodi and Maranas (2012) developed a community FBA modeling framework and applied it to a number of systems, including those of Stolyar et al. (2007) and Taffs et al. (2009). A novel aspect in this work is the consideration of multiple objective functions, including maximization of growth of each species as well as biomass production at the community level, which can be used to explore tradeoffs between selfish and altruistic driving forces.

Other multi-species, directly linked metabolic flux models were produced by Taffs et al. (2009), Bizukojc et al. (2010), Bordbar et al. (2010), Freilich et al. (2011), Khandelwal et al. (2013), Nagarajan et al. (2013), Shoaie et al. (2013), Ye et al. (2014), El-Semman et al. (2014), Merino et al. (2015) and Heinken and Thiele (2015).

##### Aggregated models

Taffs et al. (2009) applied different approaches to model three species (oxygenic phototrophs, filamentous anoxygenic phototrophs and sulfate-reducing bacteria) in the thermophilic, phototrophic mat communities from Octopus and Mushroom Springs in Yellowstone National Park (USA). One of their approaches does not consider compartments, but lumps all reactions into one species (see Box 1). This approach ignores compartmentalization and the fact that intermediate intracellular metabolites from one species may not be available to another. However, it does not require assigning individual enzymes or reactions to species, functional groups or guilds and is well suited for data from metagenomics. A unique aspect of this study is the use of elementary mode analysis (EMA), which is an alternative to FBA and characterizes the set of all possible flux distributions, rather than just the optimal one.

Tobalina et al. (2015) applied the aggregated approach to naphthalene-contaminated soil communities. An interesting aspect of that study was that the model was based on metaproteomics data, which implicitly accounts for regulation.

Cerqueda-García and Falcón (2016) applied the aggregated approach to study the metabolism of communities in microbial mats and microbialites (living carbonate rock structures similar to corals and stromatolites). Starting with metagenomic datasets, they reconstructed a metabolic network, and then used EMA to identify feasible pathways through this network for C and N assimilation. They identified a number of alternative CO_2_ fixation pathways, which were not identified for these systems previously.

#### Strengths

- The FBA approach can directly utilize molecular data, genomics, transcriptomics, proteomics and metabolomics, from pure laboratory cultures and the environment (e.g., metagenomics) as long as annotations are available, which is increasingly the case.
- The approach is comprehensive in terms of functions and metabolites. This is likely to be increasingly useful, as recent observations from a number of environments suggest that bacteria have a high substrate specificity (Kindaichi et al., 2013; Salcher et al., 2013). For example, when a freshwater community was presented with 14 radiolabeled low-molecular weight (LMW) organic substrates, the two most abundant microbes belonging to the *Actinobacteria* ac1 and *Alphaproteobacteria* LD12 tribes had no overlap in their substrate acquisition spectra. The concept of dissolved organic carbon (DOC) as a common currency for heterotrophic microbes is too simplistic. One of the main applications of FBA has been to understand complex substrate uptake patterns.

#### Weaknesses

- The directly linked and aggregated approaches assume the system to be in a steady-state. The environmentally coupled approach also assumes steady-state flux distributions during each time step, but flux distributions can change from time to time. For many cases this assumption will be sensible, but for others not. For example, planktonic bacteria experience a very heterogeneous nutrient regime and may experience nutrient patches with short durations (~60 s, Taylor and Stocker, 2012), comparable to the time required for gene expression, protein translation and maturation. Genome-scale models are being developed that go beyond steady-state metabolite fluxes (e.g., include dynamic transcript, protein and metabolite pools, Karr et al., 2012) and this technology will eventually be applied at the ecosystem scale.
- It is not always clear what objective function should be used to optimize the flux distribution (Schuster et al., 2008). Maximization of biomass production seems like a good choice from a biotechnological perspective. However, there are cases where it is advantageous to divert production away from biomass, including to storage products, toxins or EPS (Merino et al., 2015), which may conflict with the biomass objective. Moreover, in a well-mixed, stable environment, specific growth rate will likely be maximized by natural selection while in a spatially structured environment such as a biofilm, the biomass yield is likely to be maximized by natural selection (Kreft, 2004).
- The approach typically entails specifying a biomass composition, and commonly this is applied across different conditions. However, the biomass composition is known to change (Benyamini et al., 2010).
- Growth dilution of metabolites, other than the ones used in the growth equation (see above), is typically ignored (Benyamini et al., 2010). Specifically, there should be a “–μ *x*” on the right-hand side of Equation B1.1. Accounting for growth dilution is conceptually straightforward but it requires specifying the metabolite concentrations, which are not typically available at the genome scale. Metabolomics data can help to fill this gap, but this would be difficult for all metabolites, times and locations in the model and impossible for prediction simulations. Another hurdle is the computational cost. The metabolite dilution FBA (MD-FBA) model of Benyamini et al. (2010) uses mixed-integer linear programming (MILP, vs. LP used by FBA), which is computationally more demanding than LP. This limitation may be especially important for applications that require solutions for multiple species, times and locations.
- The approach does not account for individual heterogeneity (see Part 2).

### Gene-centric modeling

#### Definition

In the gene-centric or functional gene approach, the model is built based on genetic information, as in metabolic flux modeling, but focused on capturing the dynamic behavior of specific genes or gene activities in the system. Thus, the biogeochemical fluxes are based on the genetic composition of the microbial community. Microbes are grouped based on specific functional or proxy genes and tracked using corresponding concentration state variables. This is similar in spirit to modeling functional groups (e.g., N-fixers, lactate producers, Le Quéré et al., 2005; Kettle et al., 2015). The concentrations of genes (e.g., number of gene copies per liter) are simulated using mass balance differential equations, which is how typical microbial ecology models simulate species. The rate of gene production (or growth) can be tied to the Gibbs free energy released by the reaction catalyzed by the corresponding enzyme. The approach is illustrated in Box 2.

Box 2 **From genes to ecosystems using gene centric modeling.**

**Figure B2:**
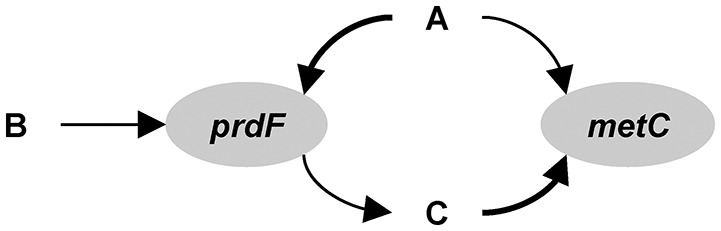
Gene centric modeling.

The following description is based on Reed et al. (2014), but adapted to the hypothetical ecosystem considered here and some of the nomenclature is altered to facilitate comparison with the other approaches. The first step in the development of the model is to identify the functional genes. For the hypothetical example, we will use *prdF* and *metC* and ignore the others (Figure B2). Thus, state variables *prdF*_*g*_ and *metC*_*g*_ (no. L^−1^) represent species 1 and 2, respectively. The reactions mediated by these gene products are assumed to be the limiting reactions along the pathway, but exchange with extracellular metabolites requires accounting for the input and output of the entire pathway. For *prdF*, the overall reaction is:

(B2.1) $$\begin{array}{l} A^{ext}+2\;B^{ext}\;\overset{prodF}{\to }\;2\;C^{ext} \end{array}$$

The production rate of *metC* genes as a result of metabolism associated with the *metC* gene (*R*_*metC*_, no. L^−1^ d^−1^) is:

(B2.2) $$\begin{array}{l} R_{metC}=metC_{g}\;F_{T,metC}\;\mu_{max,metC}\;\frac{C_{C}}{K_{m,metC,C}+C_{C}} \end{array}$$

*metC*_*g*_ (no. L^−1^) is the *metC* gene concentration. *F*_*T,metC*_ is a thermodynamic potential factor, which accounts for the chemical energy available to drive the metabolism, and can be estimated from the energy yield of the associated reaction. μ_*max,metC*_ (d^−1^) is the maximum specific growth rate. *C*_*C*_ (molC L^−1^) and *K*_*m,metC,C*_ (molC L^−1^) are the extracellular concentration and half-saturation constant for metabolite C. The production rate of *prdF* genes (*R*_*prdF*_) is:

(B2.3) $$\begin{array}{l} R_{prdF}=prdF_{g}\;F_{T,prdF}\;\mu_{max,prdF}\;\frac{C_{A}}{K_{m,prdF,A}+C_{A}}\;\frac{C_{B}}{K_{m,prdF,B}+C_{B}}\;\frac{K_{i,prdF,C}}{K_{i,prdF,C}+C_{C}} \end{array}$$

The last fraction accounts for inhibition by metabolite C. The mass balance equation for extracellular metabolite B is (transport and other reactions are omitted for clarity):

(B2.4) $$\begin{array}{l} \frac{dC_{B}}{dt}=-\frac{\gamma_{prdF,B}}{\gamma_{prdF,A}}\frac{R_{prdF}}{q_{prdF}\;Y_{prdF}} \end{array}$$

γ_*prdF,B*_ and γ_*prdF,A*_ are stoichiometric coefficients. Here, 2 mol B are consumed for every 1 mol A, so γ_*prdF,B*_ = 2 and γ_*prdF,A*_ = 1. *q*_*prdF*_ (no. gDW^−1^, i.e., per gram biomass dry weight) is the intracellular concentration of *prdF* genes, which depends on the number of gene copies in the genome. *Y*_*prdF*_ (gDW molA^−1^) is the yield, which depends on the energy yield of the associated reaction. For metabolite A, we have to consider consumption by both species:

(B2.5) $$\begin{array}{l} \frac{dC_{A}}{dt}=-\frac{R_{prdF}}{q_{prdF}\;Y_{prdF}}-\frac{\gamma_{metC,A}}{\gamma_{metC,C}}\frac{R_{metC}}{q_{metC}\;Y_{metC}} \end{array}$$

Here, A and C are consumed in equal amounts, so γ_*metC,A*_ = γ_*smetC,C*_ = 1. Metabolite C is consumed by the reaction associated with *metC* and produced by the reaction associated with *prdF*:

(B2.6) $$\begin{array}{l} \frac{dC_{C}}{dt}=-\frac{R_{metC}}{q_{metC}\;Y_{metC}}+\frac{\gamma_{prdF,C}}{\gamma_{prdF,A}}\frac{R_{prdF}}{q_{prdF}\;Y_{prdF}} \end{array}$$

Here, 2 mol C are produced for every 1 mol A consumed, so γ_*prdF,C*_ = 2 and γ_*prdF,A*_ = 1. The mass balance for gene *prdF* is:

(B2.7) $$\begin{array}{l} \frac{dprdF_{g}}{dt}=R_{prdF}-k_{d}prdF_{g} \end{array}$$

where *k*_*d*_ (d^−1^) is the death rate. The first term in the gene mass balance equation accounts for the production of the gene due to the growth associated with its reaction and the second term accounts for mortality.

#### Examples

**Figure 3:**
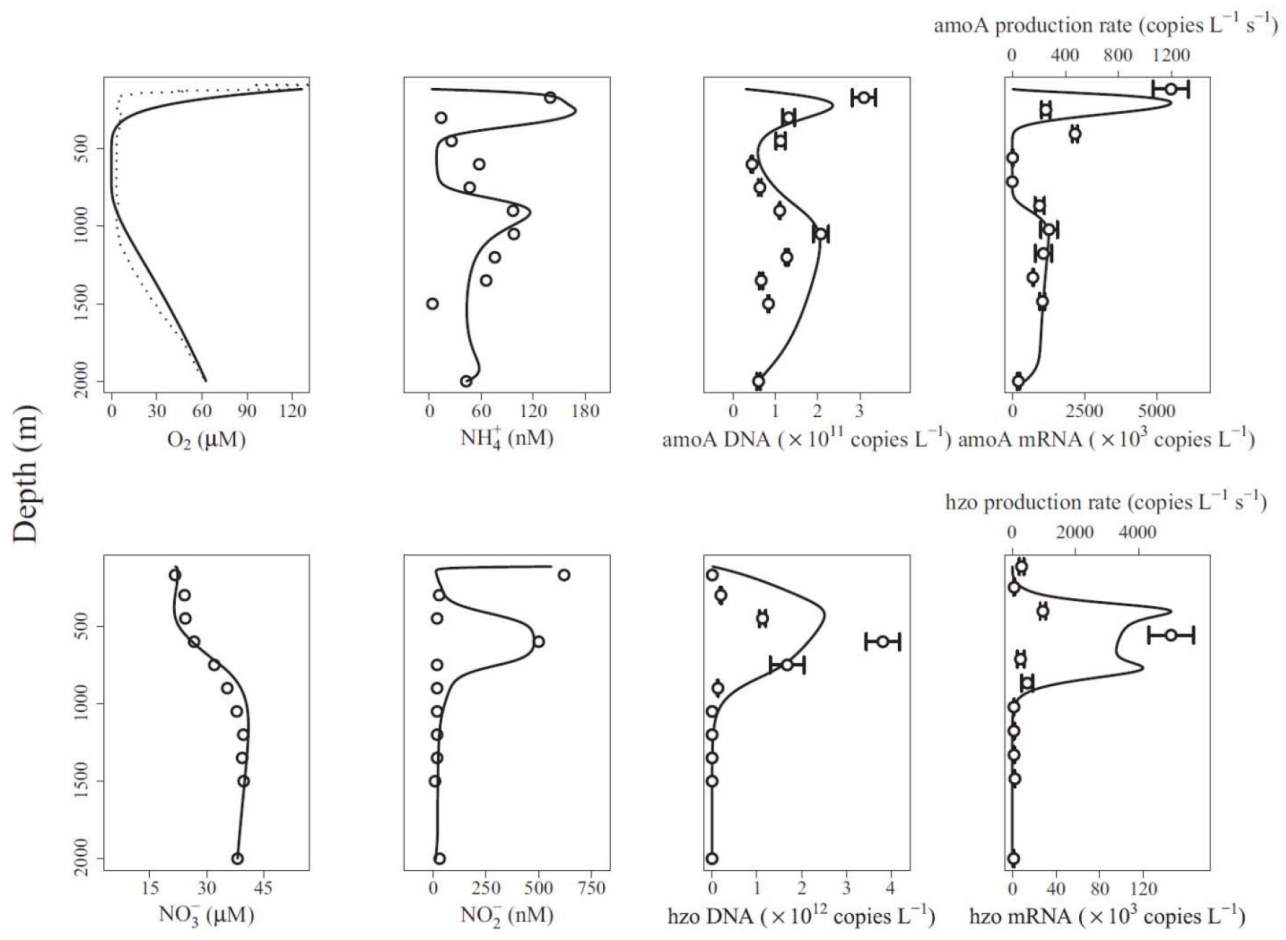
Comparison of gene-centric model predictions (solid lines) to observations (dotted line in top left and symbols in other panels) of metabolites, gene and mRNA levels in the Arabian Sea oxygen minimum zone. From Reed et al. (2014). Copyright © (2014) by the National Academy of Sciences. The figure shows an oxygen low from about 400 to 900 m and a coincident low in ammonia monooxygenase (*amoA*) DNA and mRNA, and an increase in anaerobic ammonium oxidation (anammox, hydrazine oxidoreductase, *hzo*) DNA and mRNA.

Reed et al. (2014) presented the gene-centric approach and applied it to study nitrogen cycling in the Arabian Sea oxygen minimum zone (OMZ). The model includes eight functional genes, including those for denitrification (nitrate reductase, *narG*, nitrite reductase, *nirK*), aerobic ammonia oxidation (ammonia monooxygenase, *amoA*) and anaerobic ammonium oxidation (anammox, hydrazine oxidoreductase, *hzo*), as well as relevant metabolites, including dissolved oxygen (O_2_), ammonium (${\text{NH}}_{4}^{+}$), nitrate (${\text{NO}}_{3}^{-}$), and nitrite (${\text{NO}}_{2}^{-}$). The model-predicted gene abundances were compared directly to observations from qPCR (gene copies L^−1^, Figure 3). The authors also compared model-predicted changes in gene abundances over time to observed mRNA concentrations in a qualitative manner (gene copies L^−1^ s^−1^ vs. mRNA copies L^−1^, Figure 3). An interesting problem addressed by this model is the dual role of nitrogen as an energy source and biomass component, where the latter is not considered by the gene-centric approach. This was handled by calculating the total biomass increase/decrease and removing/adding corresponding amounts of N from/to the extracellular metabolite pools. The model was used to show that denitrification is the dominant nitrogen loss process in this area, which is different from many other OMZs, where anammox dominates.

Reed et al. (2015) applied the gene-centric approach to simulate functional genes for sulfur, nitrite, ammonia, methane and hydrogen oxidation and associated metabolites in a submarine hydrothermal vent plume. The pathways for oxidation of a number of reduced sulfur species (e.g., hydrogen sulfide, thiosulphate) co-occur in one species (SUP05), so the pathways were combined into one functional gene in the model. The authors compared their model-predicted relative abundance (%) of functional genes to observations. The hydrogen concentration is relatively low in this system and their model predicted no significant increase in hydrogenase gene abundance due to aerobic hydrogen oxidation. However, substantial quantities of hydrogenase genes were observed suggesting that they may be produced because they co-occur with a gene that does experience substantial growth as observed (SUP05 may have genes for sulfur and hydrogen oxidation, Anantharaman et al., 2013). When this coupling is included in the model, it was able to reproduce the observations.

Louca et al. (2016) presented a gene-centric model of six functional genes and eight metabolites for a number of dissimilatory redox pathways involved in nitrogen and sulfur cycling in a seasonally anoxic fjord (Saanich Inlet, Vancouver Island, Canada). That model extends the gene-centric modeling approach by explicitly simulating mRNA and proteins, assuming their production rates are proportional to the corresponding reaction rates and subjecting them to transport and decay processes. Model predictions for mRNA and proteins were compared to observations on a qualitative basis. The model was used to gain insights into the sulfur and nitrogen pathways in this system. For example, the model predicted incomplete denitrification by the SUP05 clade, which results in leakage of nitrite that supports anammox and loss of nitrogen.

#### Strengths

- The approach is readily integrated into existing models based on concentration state variables (Reed et al., 2014).
- The approach makes quantitative predictions of gene levels that can be compared directly to observations.

#### Weaknesses

- While this modeling approach is readily applied to chemotrophs where there is a direct link between the rate of the reaction and the growth rate of the microbe, it is less clear how to apply it to a phytoplankton species that may be limited by nitrate, but uses the energy derived from sunlight to reduce it to ammonia for incorporation into amino acids. Heterotrophs growing on a complex mixture of dissolved organic matter (DOM) may also be difficult to model with this approach.
- The extension of the method to mRNA and proteins (Louca et al., 2016) includes simulating them as independent concentration variables. This does not account for their natural co-existence in the cell and may lead to some odd effects, like mRNA and protein appearing in locations where there are no corresponding genes.
- The method supports multiple co-occurring genes (see Reed et al., 2014 for equations), but that is based on constant fractions within a community, which may change dynamically and spatially in a natural community (e.g., species succession in phytoplankton). This method is also more difficult to implement. The reader is invited to rework the example in Box 2 for a model that uses *metA* and *metC* (which co-occur in species 1) as functional genes.
- The approach does not account for individual heterogeneity (see Part 2).

### Agent-based modeling (ABM)

#### Definition

ABM or individual-based modeling (IBM) involves simulating microbes as individuals. This is in contrast to the traditional population-level approach, where microbes are simulated as concentration state variables. ABM is already an established modeling technology in microbial ecology (Hellweger et al., 2016a). Microbial ABMs increasingly resolve intracellular mechanisms and the extension to genes is a natural progression and already well on the way. The approach is illustrated in Box 3. This approach has also been referred to as Systems BioEcology (Hellweger, 2009).

Box 3 **From genes to ecosystems using agent-based modeling.**

**Figure B3:**
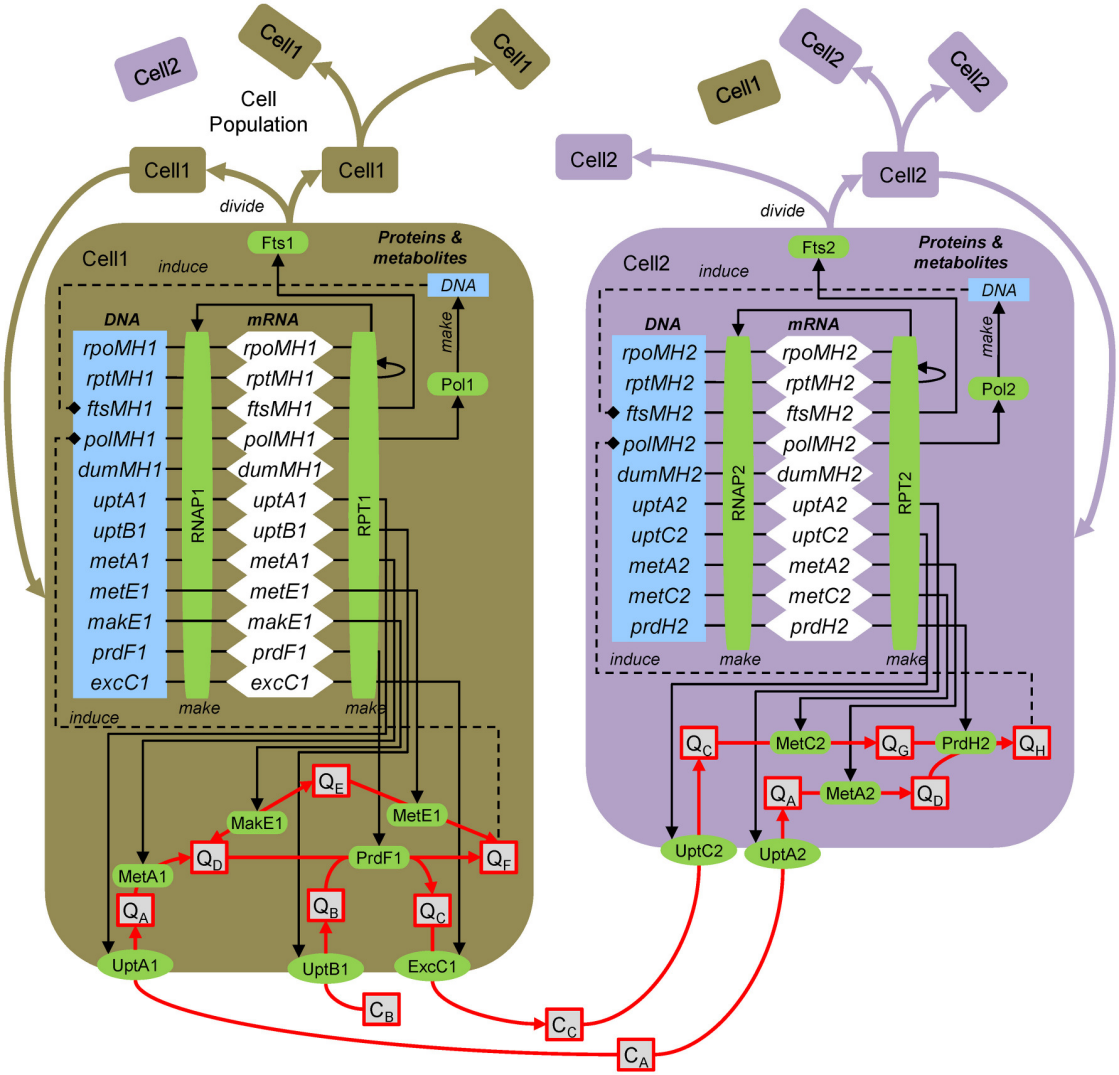
Agent-based modeling of microbes. Gene/protein: *rpoMH*/RNAP, RNA polymerase; *rptMH*/RPT, ribosome; *ftsMH*/Fts, cell division; *polMH*/Pol, DNA polymerase; *dumMH*, dummy (accounts for genes not explicitly considered); *uptA*/UptA, uptake A; *metA*/MetA, metabolism A; *excC*/ExcC, excretion C. Substrates and metabolites (extracellular, concentration C; intracellular, quota Q): C_A_, substrate A; Q_H_, metabolite H, etc. After Figure 1 of Hellweger (2009).

Here the approach for dynamic, molecular-level, mechanistic modeling is illustrated by application to the hypothetical ecosystem (Figure 1). The model explicitly resolves genes, transcripts, proteins and metabolites (Figure B3). Following the central dogma of biology, genes are transcribed by the RNA polymerase to yield transcripts (mRNA), which are translated by the ribosomes to yield proteins, which then carry out various functions. Once a biomass (in the case of strain 1 the metabolite E, Q_E_) threshold is reached, the DNA polymerase is induced, which synthesizes DNA. Once that is complete, cell division is induced and the cell divides, which involves division of all intracellular components. The approach entails explicitly modeling genes, transcripts and proteins. However, typically only a handful of representative genes are simulated using a coarse-grained approach (Castellanos et al., 2004; Hellweger, 2013).

To illustrate the approach, we present the equations for *uptB1* transcription, UptB1 synthesis, UptB1 rate and Q_B_ mass balance. Here, intracellular concentrations are defined on a per biomass dry weight (DW) basis, but carbon and volume can also be used. The *uptB1* transcript (*uptB1*_*t*_, mol mRNA gDW^−1^, i.e., per gram biomass dry weight) mass balance equation is:

(B3.1) $$\begin{array}{l} \frac{duptB1_{t}}{dt}=\frac{k_{S,T}}{L_{DNA}}RNAP1\;\gamma_{uptB1}uptB1_{g}-k_{d,T}uptB1_{t}-\mu_{g}uptB1_{t} \end{array}$$

where *k*_*S,T*_ (bp RNAP^−1^ s^−1^) is the transcription rate, *L*_*DNA*_ (bp) is the total DNA length, *RNAP1* (mol protein gDW^−1^) is the RNA polymerase level, *y*_*uptB*1_ is the *uptB1* expression level (may depend on various factors), *uptB1*_*g*_ is the number of *uptB1* gene copies, *k*_*d,T*_ (s^−1^) is the mRNA decay rate and μ_*g*_ (s^−1^) is the specific growth rate. The UptB1 (mol gDW^−1^) protein mass balance is:

(B3.2) $$\begin{array}{l} \frac{dUptB1}{dt}=k_{S,P}\;\frac{uptB1_{t}}{TxL}RPT1-k_{d,P,UptB1}UptB1-\mu_{G}UptB1 \end{array}$$

where *k*_*S,P*_ (nt RPT^−1^ s^−1^) is the translation rate, *TxL* (mol mRNA gDW^−1^) is the total mRNA, *RPT1* (nmol protein gDW^−1^) is the ribosome level and *k*_*d,P,UptB*1_ (s^−1^) is the UptB1 decay rate. The UptB1 rate (*V*_*UptB*1_, mol gDW^−1^ s^−1^) is:

(B3.3) $$\begin{array}{l} V_{UptB1}=UptB1\;k_{UptB1}\frac{C_{B}}{K_{m,UptB1}+C_{B}}\frac{K_{i,UptB1}}{K_{i,UptB1}+Q_{B}} \end{array}$$

where *k*_*UptB*1_ (molB molUptB1^−1^ s^−1^) is the UptB1 catalytic rate constant, *K*_*m,UptB*1_ (molB L^−1^) is the half-saturation constant and *K*_*i,UptB*1_ (molB gDW^−1^) is the inhibition constant. The intracellular metabolite B (*Q*_*B*_, mol gDW^−1^) mass balance is:

(B3.4) $$\begin{array}{l} \frac{dQ_{B}}{dt}=V_{UptB1}-2\;V_{SynF1}-\mu_{G}\;Q_{B} \end{array}$$

where *V*_*PrdF*1_ (molF gDW^−1^ s^−1^) is the PrdF1 reaction rate (the factor 2 accounts for 2 mol B per 1 mol F, see Figure 1).

#### Examples

ABM was used by Hellweger (2009) to explore the role of photosynthesis genes (*psbA, hli*) carried by viruses that infect the marine cyanobacterium *Prochlorococcus*. The idea is that these genes help to maintain the host photosynthesis apparatus during the latent period, increasing energy to support the replication of the virus. The model simulates individual viruses and host cells and explicitly resolves mechanisms of gene expression, protein synthesis, photosynthesis and events associated with infection at the molecular level. The model was calibrated to observations of virus and host gene transcript and protein levels and then used to simulate population dynamics in the water column of the Sargasso Sea. Modeled populations were diverse, including multiple virus types (different combinations of *psbA* and *hli* copies) and cells with different light histories, cell cycle phases and infection stages. Using competition experiments between virus strains that have different combinations of *psbA* and *hli*, and evolution experiments (i.e., gene packaging error), the model predicted an optimal gene content that matched that of the wild-type.

An ABM of the cyanobacterium *Synechococcus* and its circadian clock was constructed by Hellweger (2010). The model structure is similar to the *Prochlorococcus* model described above. A new feature was the explicit simulation of the concentration of proteins with different phosphorylation states and their interaction. The modeled population includes cells at different phases in their cell and circadian cycles and gene expression levels (*psbAI* luminescence) were compared to observations at the individual level.

Mina et al. (2013) used an ABM of genetically-engineered quorum sensing *E. coli* cells in a three-dimensional microfluidics chamber. The model simulates a heterogeneous population of individual, motile cells, each with a number of genes (*luxI, aiiA*, and *yemGFP*) and associated proteins, which communicate via a diffusible substance. They showed that autoinducer oscillations on the population level do not follow simply from synchronizing single cell oscillations. Single cells can switch between a state of constant signal concentration and oscillations, depending on the parameters of the positive and negative feedback loops in the gene regulatory network. Yet in a population of these cells, only the oscillatory state is stable—once cell density exceeds a threshold.

Hellweger et al. (2014) built an ABM of the yeast *Saccharomyces cerevisiae* and used it to explore the fitness effect of age-correlated stress resistance. The model explicitly simulates the regulation of the proteins Tsl1 and Tps3, which synthesize the stress protectant trehalose. Their expression is modeled using constant, age-dependent and stochastic terms. The population is diverse consisting of cells in different phases of their cell cycles as well as different ages, damage and Tsl1/Tsl3 expression levels. The modeled heterogeneity was compared to observations obtained using flow cytometry. Comparison of the various expression strategies showed that age-correlated stress resistance can be beneficial under some conditions.

**Figure 4:**
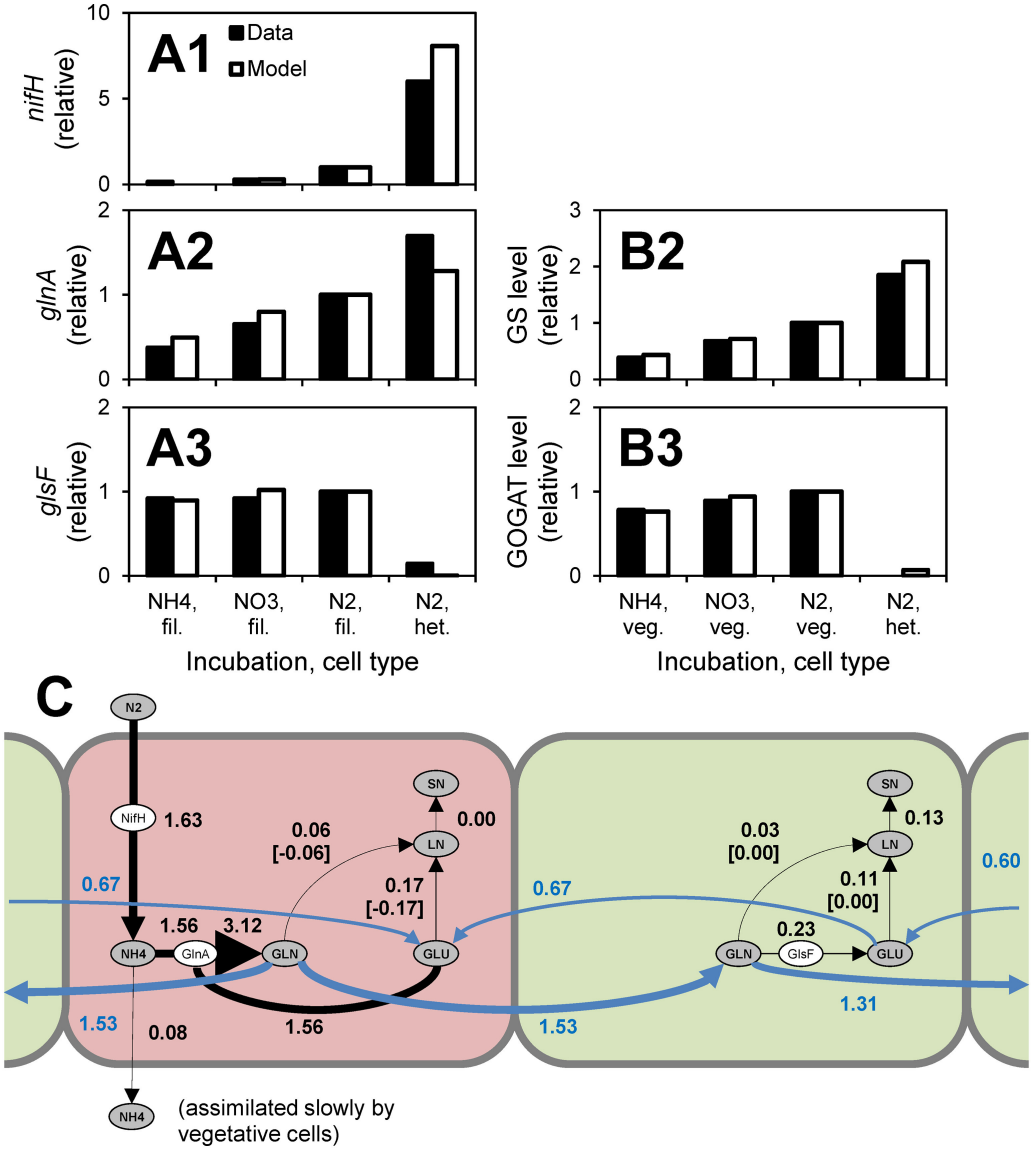
*Anabaena*—nitrogen interaction model. **(A,B)** Comparison to observations of transcript levels in filaments (fil., i.e., all cells), vegetative cells (veg.) and heterocysts (het.) **(A)** and enzyme activities **(B)** of cells grown under different conditions (data are from Martin-Figueroa et al., 2000). **(C)** N fluxes for growth on N_2_. Red, heterocysts; Green, vegetative cells. Numbers are fluxes in pmol N cell^−1^ d^−1^. From Hellweger et al. (2016b). The figure shows that heterocysts have higher levels of nitrogenase (*nifH*) transcripts, higher levels of glutamine synthetase (*glnA*, GS) transcripts and enzyme levels and lower levels of glutamate synthase (*glsF*, GOGAT) transcripts and enzyme activities. These observations support the model where N_2_ is fixed in heterocysts and combined with glutamate (GLU) that is imported from adjacent vegetative cells to yield glutamine (GLU), which is then exported to vegetative cells and further incorporated into labile nitrogen (LN) and structural nitrogen (SN) pools.

A model of *Anabaena*—nitrogen interactions was developed by Hellweger et al. (2016b). This model simulates the uptake of various forms of nitrogen and early intracellular assimilation pathways. Uptake and intracellular reactions are mediated by enzymes (e.g., GlnA) and their expression is controlled by a number of regulatory proteins (e.g., NtcA). A novel feature of this model is the explicit simulation of cell differentiation and division of labor. When fixed nitrogen is depleted, the cells become nitrogen-stressed and some differentiate into heterocysts, which are anoxic cells that fix nitrogen and pass the fixed nitrogen to their neighboring vegetative cells (Figure 4C). The model was informed by observations from 269 laboratory experiments from 55 papers published from 1942 to 2014, including transcript levels and enzyme activities (Figures 4A,B). Hellweger et al. (2016b) also applied the model to a hypothetical lake, but validation by comparison to field observations was not performed.

Another gene-level ABM was developed by Hellweger (2013) to explore the mechanisms underlying adaptation of *E. coli* to tetracycline resistance.

#### Strengths

- The main advantage of ABM is the ability to resolve intra-population heterogeneity. We will discuss the importance of individuality in the second part of this review.
- In ABM, the description of the system is very flexible and not constrained by having to use one specific mathematical formalism (e.g., the stoichiometric matrix of the FBA approach). For example, on/off control of a gene by light can be modeled simply using an “if” statement (if light is on, then turn on gene, otherwise turn it off). It is much more difficult to incorporate this into a stoichiometric matrix or differential mass balance equation.

#### Weaknesses

- This approach is relatively complex and difficult to apply. Although it can theoretically be extended to the whole-genome scale, past models have focused on a handful of genes, transcripts, proteins and metabolites. This is due to the limited availability of rate formulations and parameters, and the difficulty of calibrating a model with numerous non-linear feedbacks.

### Summary of examples

**Figure 5:**
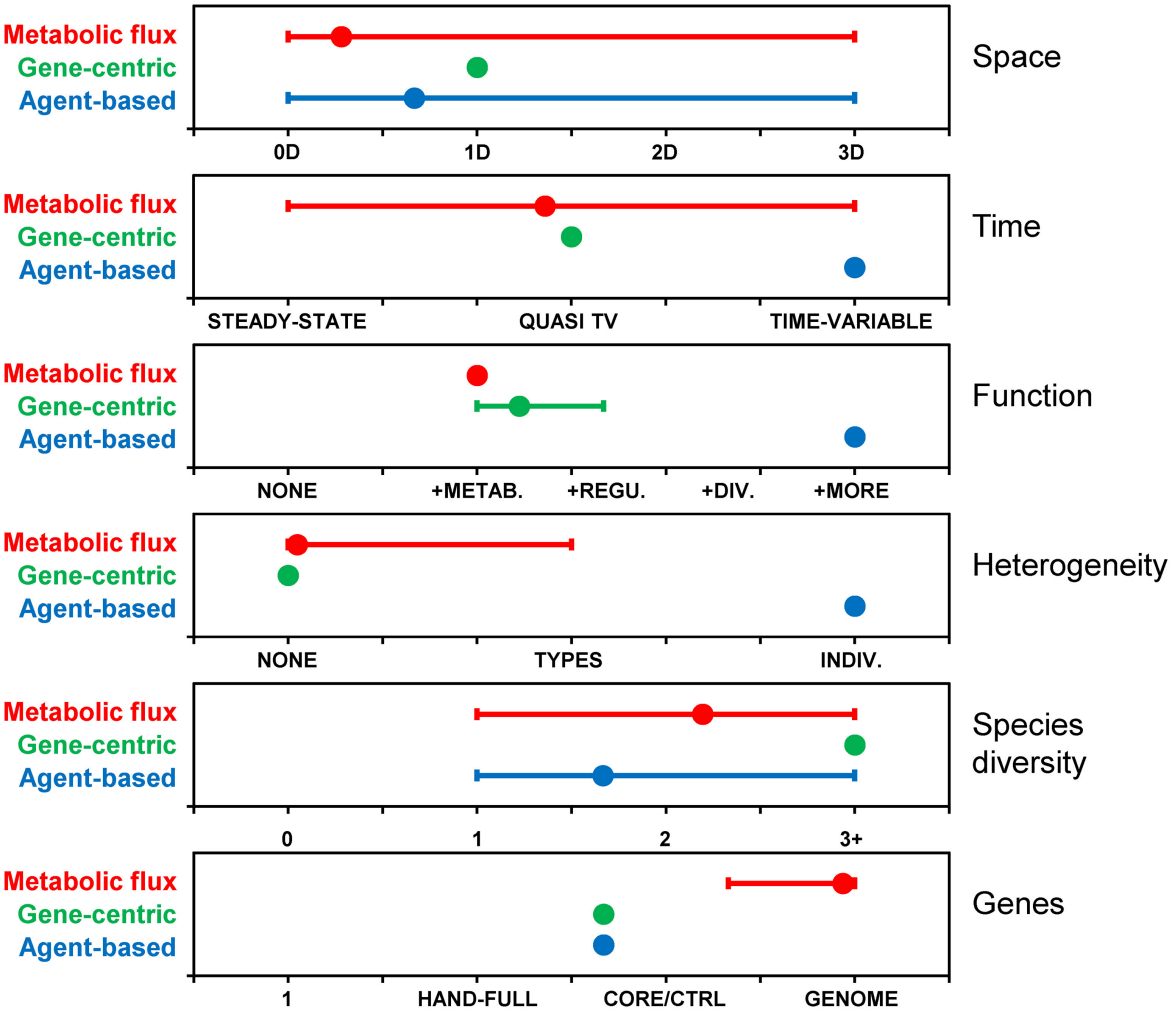
Comparison of modeling approaches along various dimensions. Based on metabolic flux (*N* = 32), gene-centric (*N* = 3) and agent-based (*N* = 6) models included in this review. Dimensions: Space: 0 (i.e., well-mixed reactor), 1, 2 or 3 dimensional; Time: steady-state, quasi-time-variable (e.g., dynamic FBA), time-variable; Function: None, metabolism, + regulation, + division, + additional functions; Heterogeneity (individuality): None, types (i.e., phenotypes), individuals; Species diversity: None, one, two, three or more; Genes: None, a handful, core/central metabolism, whole genome. Symbols represent averages and “error bars” the range between minimum (i.e., dimensions covered by all models) and maximum. For example, agent-based models have been developed with zero to three spatial dimensions, and the average is 0.67.

The models reviewed above are characterized along six different dimensions, including space, time, function, heterogeneity, species diversity, and genes (Figure 5). These dimensions were selected as they highlight differences between the reviewed models, nevertheless, the list is not exhaustive and other dimensions can be used, like types of interactions. The figure illustrates that, together, the population of past models covers the entire space. However, no single model or approach has covered the entire space by itself. The gene centric approach is amenable to space, time and species diversity and those dimensions have been explored in past models. Function and genes dimensions are linked in this approach, and limited because each species is typically associated with only one function. Simulating individual heterogeneity is difficult with the population-level gene centric approach and has not been explored in past models. Metabolic flux models are routinely genome-scale, and past models have pushed the boundaries along other dimensions, including space, time (although only quasi-time-variable), and species diversity. Function is often limited to metabolism and heterogeneity to phenotypes. The agent-based approach is flexible along most dimensions, but limited in terms of gene coverage and models with more than a handful of genes have yet to be developed.

## Part 2: the importance of individuality

A key distinction between the modeling approaches reviewed above is their consideration of individuality and heterogeneity. It is now well established that microbial populations in the environment and laboratory exhibit substantial heterogeneity in properties and behaviors. There are probably cases where this individuality averages out and is of no consequence to the ecology or biogeochemistry of the system (e.g., steady-state growth of a single species on a single nutrient). However, there are also cases where individuality has been shown to critically affect the fitness of a population. A thorough understanding of individual heterogeneity and its potential ecological consequences is critical for selecting the most appropriate modeling strategy.

**Figure 6:**
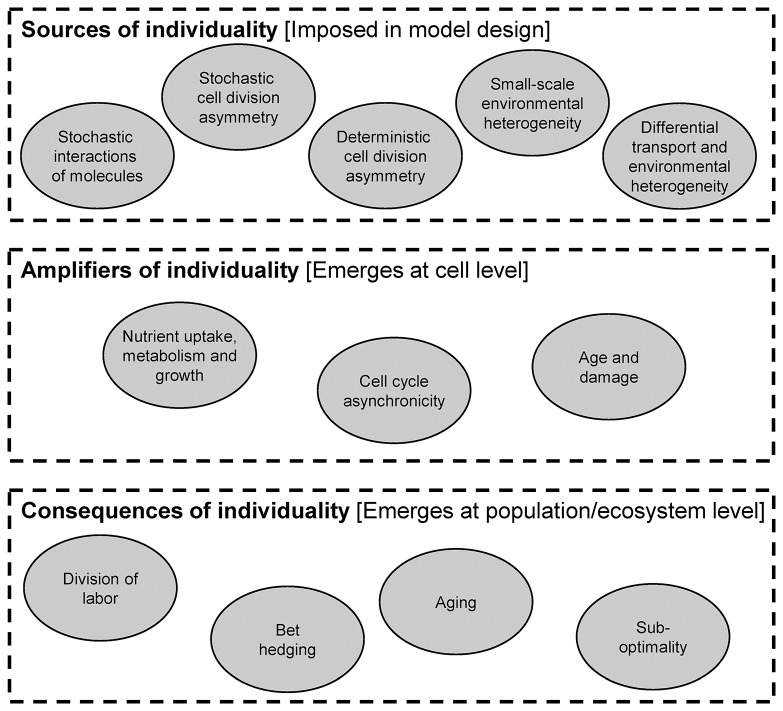
Sources, amplifiers and consequences of individual heterogeneity.

In this part, we review the importance of individuality. There are a number of mechanisms that produce heterogeneity in a population, which we refer to as sources. Once heterogeneity is introduced, it can be maintained and amplified in a number of ways. Finally, there are a number of important ecological consequences of heterogeneity (Figure 6).

From a modeling perspective, the distinction of sources, amplifiers and consequences is important. Specifically, sources of heterogeneity are included in the design of the model. In other words, there are equations or parts of the model code that produce heterogeneity. For example, stochastic cell division asymmetry can be included in an agent-based model by randomly varying the daughter biomass from the perfect 50/50 split after division. Amplifiers are mechanisms that operate at the individual level and change the cell's properties. The resulting additional heterogeneity is not prescribed in the model design, but it emerges from running it (i.e., it is a model output). For example, heterogeneity in birth sizes may lead to heterogeneity in generation times without any added equation or code. Consequences are also not included in the design of a model, but they emerge as population- or ecosystem-level properties rather than individual-level properties.

### Sources of individuality

There are many mechanisms that can produce and maintain individual heterogeneity. Here we consider a mechanism that would lead a colony growing up from a single cell to become heterogeneous to be a “source of heterogeneity.” Of course these mechanisms can also operate and produce/modify heterogeneity in other scenarios.

#### Stochastic interactions of molecules

Intracellular “concentrations” of transcription factors and macromolecules (DNA, mRNA, proteins) are often low. For example, natural populations have on average less than one transcript per gene (Cottrell and Kirchman, 2016). That means the continuum assumption underlying deterministic chemical reaction kinetics is not met and corresponding regulatory or signaling networks can exhibit substantial stochasticity. This leads to intra-population heterogeneity and when coupled with positive feedbacks can lead to bi-stability and phenotypic differentiation (Veening et al., 2008). For example, the expression level of heterodisulfide reductase subunit A (central for respiration in sulfate reducers) in *Desulfovibrio vulgaris* cells varied by as much as 50-fold in a sample of 30 individual cells (Qi et al., 2014). Another example is stochasticity in the chemotaxis regulatory network. Low concentrations of signaling molecules, specifically phosphorylated CheY, lead to behavioral variability of individuals, and this can be reduced by increasing the concentration of this element in the network (Korobkova et al., 2004). However, stochastic gene expression may be more the exception than the rule as the expression of most genes in *E. coli* does not show any bursts (Silander et al., 2012). Metabolic pathways are usually considered to be unaffected by stochasticity because of the higher numbers of metabolic enzymes and metabolites in the cell, but the stochastic expression of catabolic enzymes has been found to lead to fluctuations in growth rate that can perturb the expression of other enzymes (Kiviet et al., 2014). Heterogeneity may also arise from changes to the DNA, including mutation, recombination and methylation (Avery, 2006; van der Woude, 2011).

#### Stochastic cell division asymmetry

Another source of cell-to-cell variability is stochastic partitioning of cellular components during cell division. This may be due to low copy numbers of mRNAs, proteins, plasmids and genomes (Huh and Paulsson, 2011; Jahn et al., 2015), or imperfections in the cell division machinery leading to unequal daughter cell sizes and consequently asymmetry in all cellular components. Bacteria can control this heterogeneity by molecular mechanisms that are increasingly understood. For example, interactions of MinCDE proteins with themselves and the polar membranes set up a spatial gradient inside the cell that favors assembly of the FtsZ cell division ring in the middle of the cell (Kieser and Rubin, 2014). Missing components of this regulatory system have been implicated in the higher division asymmetry observed for *Mycobacterium smegmatis* compared to *E. coli* (Aldridge et al., 2012).

#### Deterministic cell division asymmetry

**Figure 7:**
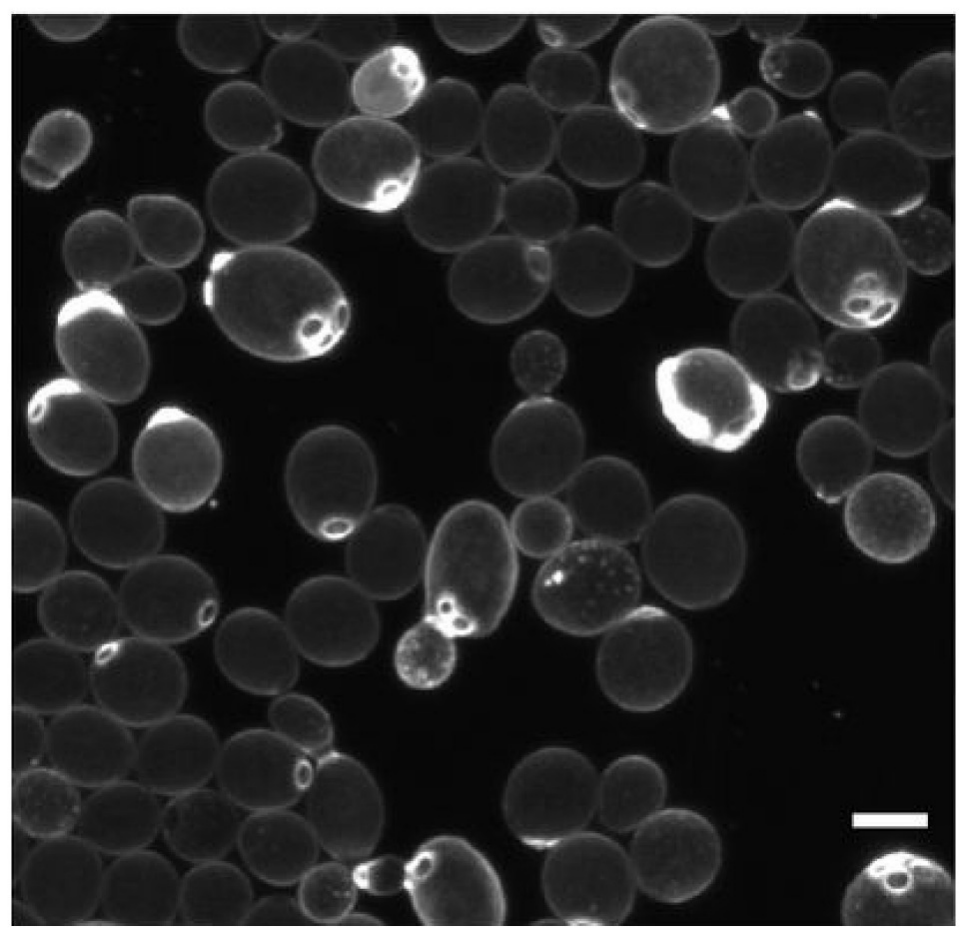
Deterministic cell division asymmetry in the budding yeast *Saccharomyces cerevisiae* leads to heterogeneity in size and bud scars (stained bright). Scale bar is 5 μm. Reproduced from van Deventer et al. (2015) with permission.

In addition to stochastic processes, there are deterministic mechanisms that lead to cell division asymmetry. Replication in budding bacteria and yeast obviously produces two different individuals and population heterogeneity. In *Saccharomyces cerevisiae*, the mother cell is larger and accumulates damage, including bud scars, extrachromosomal DNA circles (ERCs) and carbonylated proteins, which are retained preferentially by the mother cell during division by binding to special cellular compartments (Unruh et al., 2013; Figure 7). Also, for cells that divide by apparently symmetric binary fission, one “daughter” inherits the old pole and one the new pole. The old pole may have accumulated more damage or other properties over its longer lifetime. For example, division in *E. coli* is associated with asymmetric segregation of damaged protein aggregates (Lindner et al., 2008). The aggregates diffuse by stochastic Brownian motion but they are too large to enter the nucleoid region and therefore get trapped at the poles (Coquel et al., 2013). The asymmetry goes beyond damage. For example, since the outer membrane (OM) is synthesized mostly along the cylindrical part of the cell, old poles have older OM and may include proteins that were previously expressed and now repressed (Ursell et al., 2012). Bacteria with flagella at one pole will also generate two different daughter cells at division (Christen et al., 2010).

#### Small-scale environmental heterogeneity

Spatially structured microenvironments constitute another driver of heterogeneity. For example, microbes in colonies, biofilms or granular sludge flocs experience gradients in dissolved oxygen and nutrient concentrations (Wimpenny and Coombs, 1983; Matsumoto et al., 2010). The response of the microbes to these different conditions, including growth and acclimation, leads to a microbial population with heterogeneous properties. For example, the growth rate of *Pseudomonas putida* cells in biofilms was monitored using a reporter consisting of the growth rate-regulated *rrnB*P1 promoter and unstable GFP (Sternberg et al., 1999). Cells along the periphery of the biofilm were observed to grow rapidly, whereas those on the inside grew slower or not at all.

#### Differential transport and environmental heterogeneity

Transport, whether passive (e.g., with water or air) or active (e.g., chemotaxis), can act differently on individuals within a homogenous population. Water flow velocities tend to be larger near the center of conduits (e.g., pores, pipes, rivers). Even for a uniform flow field, advection is generally associated with diffusion causing individuals from one location to be transported to different locations. On leaf surfaces, cells can be dispersed to different locations and grow into microcolonies, followed by detachment and colonization elsewhere (van der Wal et al., 2013). Also, transport by active mechanisms, like chemotaxis, entails stochastic variability and can lead to different paths of individuals (due to stochastic signaling, see above) (Korobkova et al., 2004). Often the environment exhibits substantial heterogeneity at this scale. For example, the nutrient concentrations in surface waters are highly heterogeneous, with microscale patches created by lysing cells, phytoplankton exudates or marine snow (Stocker, 2012; Taylor and Stocker, 2012; Zehr et al., 2017). Similarly, nutrient availability on plant leaf surfaces varies greatly at a micrometer scale and often correlates with local topography (Remus-Emsermann et al., 2011). Even environments that are designed to be homogenous, like strongly agitated small-scale fermentors, can be heterogeneous (Dunlop and Ye, 1990). When differential transport occurs in a heterogeneous environment, it can lead to intra-population heterogeneity as microbes respond to their individual conditions (i.e., by gene expression, nutrient uptake, and growth). By the same argument, the population at any given location may be comprised of individuals with vastly different life histories (Bucci et al., 2012). For example, depending on how they were formed (by a mechanism of staying-together or coming-together), aggregates of bacteria on leaf surfaces may consist of cells that are either clonal with similar life histories or represent a variety of previous leaf surface experiences (Tecon and Leveau, 2012).

### Amplifiers of individuality

The heterogeneity produced by the above sources can manifest itself in a number of properties and behaviors, which can then feed forward and produce heterogeneity in other properties and behaviors, effectively amplifying the overall heterogeneity.

#### Nutrient uptake, metabolism, and growth

**Figure 8:**
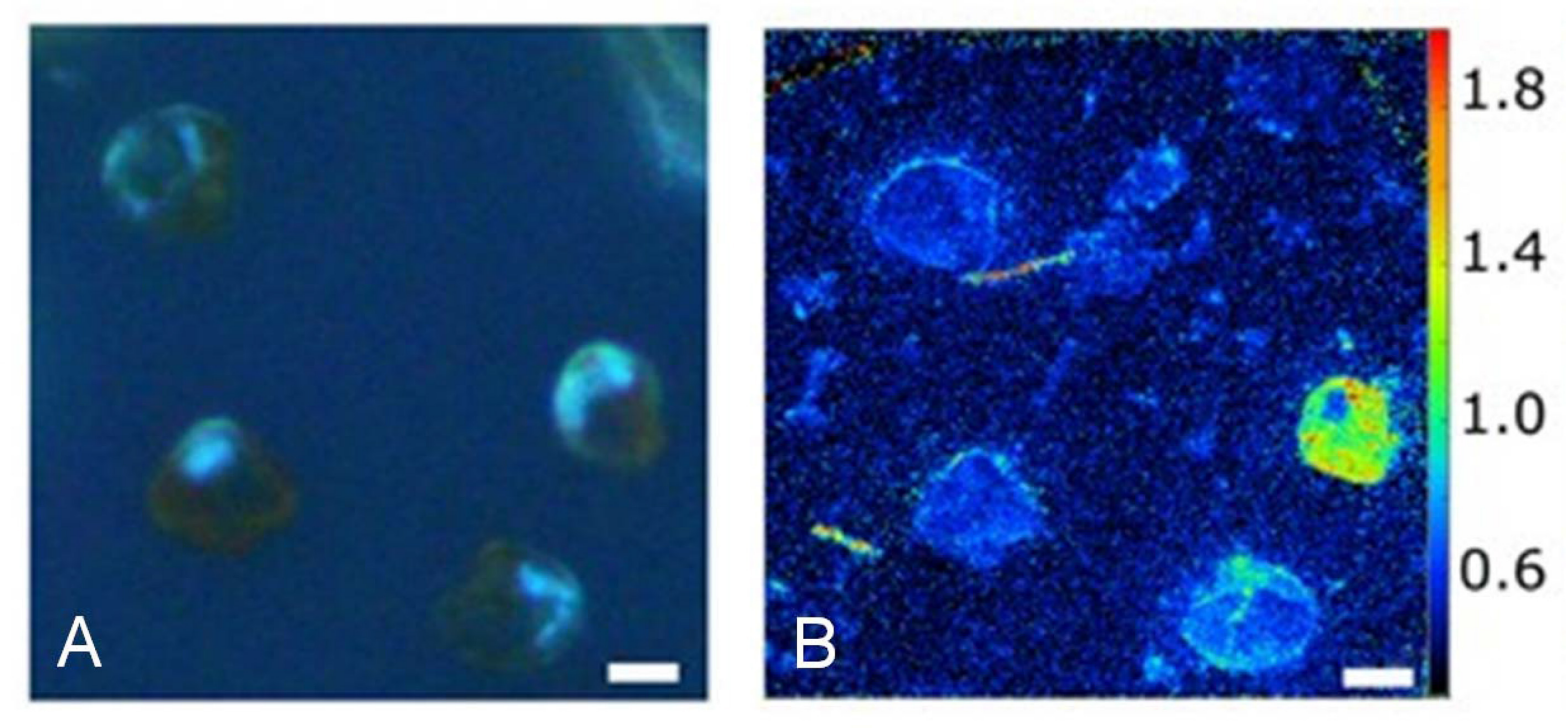
Heterogeneity in urea uptake by *P. minimum* at the single-cell level. **(A)** *P. minimum* cells in UV light. **(B)** ^15^N-urea uptake by *P. minimum* cells depicted as ^12^C^15^N^−^/^12^C^14^N^−^ ratio. Scale bar is 5 μm. Reproduced from Matantseva et al. (2016) with permission.

Stochastic gene expression, or any of the other primary sources of heterogeneity discussed above, may result in different levels of some functional enzyme and behavior, such as nutrient uptake. For example, the assimilation of nitrate and urea is very heterogeneous when a cultured population of nitrate-acclimated, marine dinoflagellate *Prorocentrum minimum* cells is exposed to a sudden input of urea (a preferred N source) (Figure 8; Matantseva et al., 2016). This may in turn affect nutrient metabolism and growth. For example, single-cell observations for *Methylobacterium extorquens* AM1 showed high variability in cell size at division, division time (2.5-fold range) and growth rate (Strovas et al., 2007). Consequently, even some bulk housekeeping functions, like metabolism and growth, which are generally considered to be relatively homogenous, can be very heterogeneous, even in cultured populations.

#### Cell cycle asynchronicity

Asymmetric division can lead to an asynchronous population (i.e., where cells are in different phases in the cell cycle) because size can be a major checkpoint for various cell cycle phases. Since the cells perform different tasks at different phases in the cell cycle, this translates into a population with heterogeneous behavior. For example, in *Saccharomyces cerevisiae*, 800 genes are cell-cycle regulated (Spellman et al., 1998) and in *Caulobacter crescentus*, over 500 genes (Laub et al., 2000). In photosynthetic microorganisms, such as microalgae and cyanobacteria, gene expression is also tied to the light-dark cycle, often via a circadian clock (Ito et al., 2009). Unless the population grows at a generation time of 1 day, it will consist of cells with various phase differences between their cell and diel cycles. This effectively adds another dimension of variation and increases the number of phenotypes and population heterogeneity.

#### Age and damage

Asymmetric segregation of damage during cell division produces younger and older cells and therefore an age distribution in the population. This affects the growth rates and other behaviors of cells. For instance, damaged protein aggregates are partitioned asymmetrically in *E. coli* and new-pole cells with less damage have a 4% higher specific growth rate (Lindner et al., 2008). In *S. cerevisiae*, older cells also grow slower and they synthesize more of the stress protectant trehalose (Levy et al., 2012).

### Ecological consequences of individuality

In many cases the heterogeneity may simply average out and be of little consequence to the fitness of the population. However, there are a number of cases where heterogeneity has been shown to have important ecological consequences.

#### Division of labor

Phenotypic differentiation forms the basis for a division of labor, where different cells carry out complementary tasks that benefit the population. For example, oxygenic photosynthesis and nitrogen fixation are incompatible processes. Specifically, the enzyme nitrogenase, encoded by genes *nifH, nifD* and *nifK* and responsible for reducing N_2_ to ${\text{NH}}_{4}^{+}$, breaks down in the presence of oxygen. To overcome this problem, the filamentous cyanobacterium *Anabaena* can differentiate into two types: photosynthesizing vegetative cells and nitrogen fixing heterocysts (Flores and Herrero, 2010). Another example includes evolved populations of *E. coli* where the labor of converting glucose to CO_2_ is divided over two cell types: one that converts glucose to acetate while the other converts acetate to CO_2_ (Harvey et al., 2014). There is also altruistic division of labor, which is the tasked sacrifice of some members of the group to benefit others. For example, *Salmonella* cells that invade the gut tissue get killed by the host immune system, but not before triggering a host response that kills other bacteria in the gut lumen but not the subpopulation of *Salmonella* cells that stayed behind and now have a competitive advantage in the gut lumen (Ackermann et al., 2008). The basis for this strategy lies in the stochastic expression of genes coding for a Type III Secretion System (T3SS) within the clonal *Salmonella* population: only a subset of cells within this population express a T3SS and it is this subset of cells that is capable of invading the gut tissue. Another example is the split into motile and immotile subpopulations of *Pseudomonas aeruginosa* that only together can generate mushroom-shaped biofilms (Ghanbari et al., 2016). Division of labor does not have to involve a direct effect of one phenotype on the other, but it may simply involve growing on different nutrients, like nitrate and urea, allowing the population to maximize uptake (Matantseva et al., 2016).

#### Bet hedging

**Figure 9:**
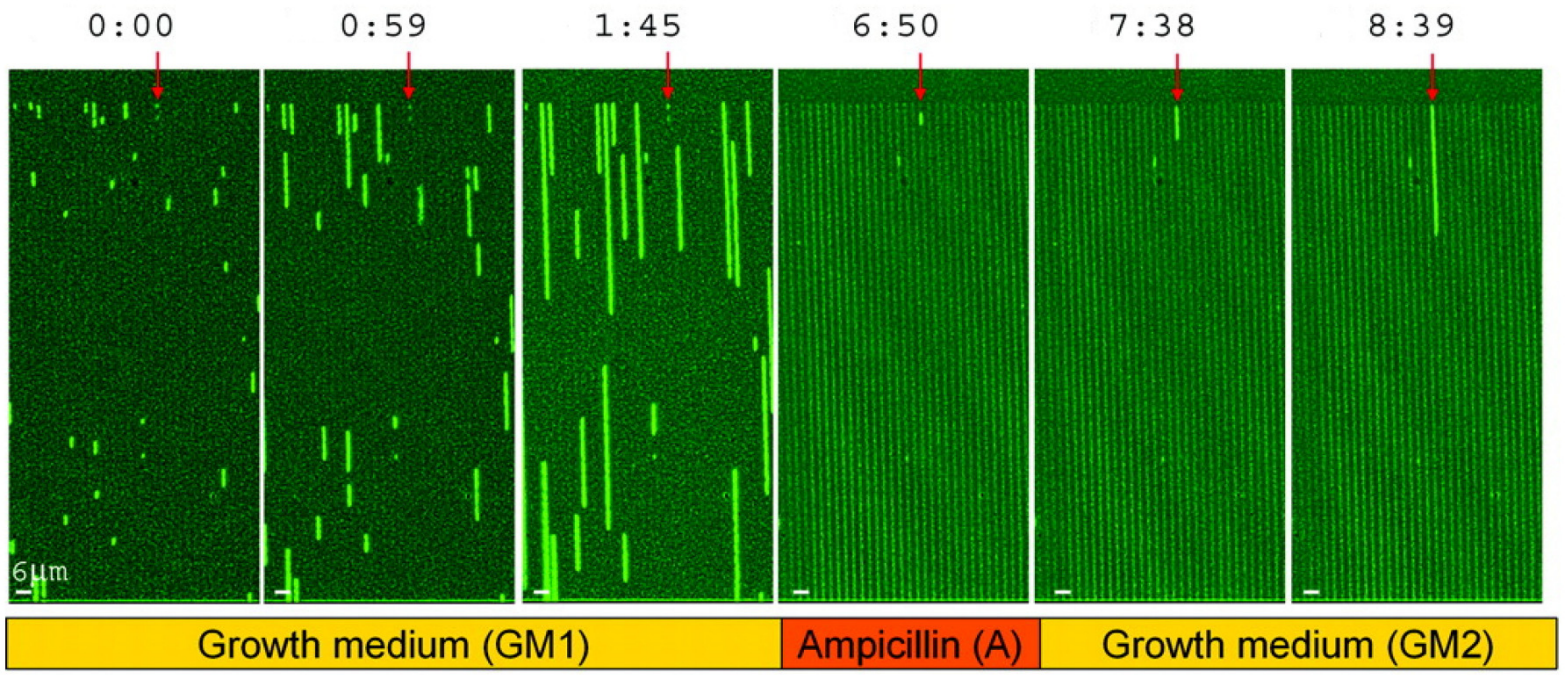
Survival of persister cells under antibiotic treatment. Growth of a hipA7 mutant, which produces a larger fraction of persisters, in microfluidic channels. Times are in hours:min. Bacteria are exposed to three phases, including growth medium (GM1), ampicillin (A) and then washing and again growth medium (GM2). Persister cells are marked with a red arrow. Reproduced from Balaban et al. (2004) with permission.

The future is uncertain and may bring unpredictable changes in stresses or any other environmental factors. If cells could react instantly to changes in their environment, a good strategy may be to rely on sensing and responding, but if the response is too slow, it is better to maintain a diversity of phenotypes (Kussell and Leibler, 2005), which is referred to as bet hedging. In the context of stress resistance, bet hedging is when a population contains some cells that are ill-adapted to the current environment but better adapted to potential future stresses. An important example are persister cells that are produced spontaneously, make up a small fraction of the population, are inhibited in growth (dormant) but can survive antibiotics (Balaban et al., 2004, 2013; Figure 9). Dormancy is widespread in microbes (Lennon and Jones, 2011), and when it involves a fraction of the cells and is not purely responsive to environmental conditions (i.e., at least partially spontaneous) it can also be considered a bet hedging strategy. For example, the cyanobacterium *Anabaena* forms akinetes that sink to the sediment bed and can serve as a seedbank for future blooms. This can be considered a bet hedging strategy to protect the population from being wiped out by some factor (washout, grazing), but it may also be a case of division of labor, as the migration to the sediment bed allows the cells to accumulate nutrients (Hellweger et al., 2008). Bet-hedging strategies do not have to involve an all-or-nothing differentiation, but can be gradual. For example, populations of the budding yeast have a gradual (and age-correlated) distribution of the stress-protectant trehalose (Levy et al., 2012). Finally, bet hedging is not restricted to stress resistance, but it may involve nutrient acquisition. For example, diversifying chemotactic behaviors in clonal populations could be an adaptation to foraging in variable environments (Frankel et al., 2014).

#### Aging

Aging is a strategy for eliminating damage from a population by concentrating it in a few cells that will eventually be discarded (i.e., die of old age). The alternative is to repair or eliminate the damage in some way. The evidence for aging to provide a significant ecological benefit in microbes is elusive, probably because the extent of damage segregation varies between species and environmental conditions. For example, for *E. coli*, one study showed asymmetric partitioning of damaged protein aggregates and decreased growth rates of older cells (Lindner et al., 2008), but in another study growth rates were not observed to decrease over many generations (Wang et al., 2010). Thus, there is an ongoing debate about the ecological benefits of aging in bacteria (Clegg et al., 2014; Koleva and Hellweger, 2015). Several recent studies suggest that aging does not increase fitness or does not occur under benign conditions but instead is a stress response at the population level (Coelho et al., 2013; Clegg et al., 2014; Iyer-Biswas et al., 2014; Vedel et al., 2016).

#### Sub-optimality

In the absence of conditions that make heterogeneity advantageous (division of labor, bet hedging or aging), it is disadvantageous or sub-optimal. For any given set of (constant) conditions, there is only one optimal behavior that maximizes fitness (for one species). This has been explored in the context of nutrient assimilation and the effect on growth. Nutrient quotas of phytoplankton can be quite heterogeneous. This heterogeneity leads to a reduction in growth rate, compared to a hypothetical population with uniform quotas, due to the non-linearity of the underlying process (Bucci et al., 2012; Fredrick et al., 2013). When the cell's environment (and thus the heterogeneity) is controlled using microfluidic culturing technology, the growth rate increases compared to flask cultures (Dusny et al., 2012). Another case of sub-optimality stems from a mismatch between cell and environmental cycles. When a population of cells in different phases of their cell cycle grows in a cyclically varying environment (i.e., diel cycle in light or temperature), the cells have different alignments between these two cycles. It is reasonable to expect that some of those alignments may be more optimal than others, leading to sub-optimality.

### Consideration of individuality in models

From the above review, it is clear that individual heterogeneity can have important effects on the ecology of microbes and the ecosystems harboring them. Any model that is to capture these effects, whether for advancing understanding or making predictions, has to be able to simulate the production and amplification of this heterogeneity. Therefore, when selecting a modeling strategy it is important to understand upfront the role of heterogeneity in the system, and how it is produced and amplified. Then, a modeling approach can be selected. For example, the ecology of infectious bacteria is in many ways controlled by bet hedging, which builds on individual heterogeneity. In some cases, such as persisters that can survive antimicrobial or other stresses, there are only two phenotypes. This type of heterogeneity has been modeled with the metabolic flux approach (Zhuang et al., 2012). Other cases involve a more gradual differentiation, like age-correlated stress resistance. Resolving this type of heterogeneity has been modeled using the agent-based approach (Hellweger et al., 2014).

## Author contributions

All authors listed have made a substantial, direct and intellectual contribution to the work, and approved it for publication.

### Conflict of interest statement

The authors declare that the research was conducted in the absence of any commercial or financial relationships that could be construed as a potential conflict of interest.
